# Prostatosymphyseal Fistula Treated by Robotic Assisted Radical Prostatectomy

**DOI:** 10.1155/2015/582705

**Published:** 2015-10-20

**Authors:** Lucy Whelan, Emma Mullarkey, Henry H. Woo

**Affiliations:** ^1^School of Medicine, University of Glasgow, Glasgow G12 8QQ, UK; ^2^Sydney Adventist Hospital Clinical School, University of Sydney, Sydney, NSW 2076, Australia

## Abstract

This case report documents a rare complication of prostate resection following a Greenlight laser procedure. The 75-year-old gentleman involved underwent photoselective vaporisation of the prostate (PVP) for clinically benign prostatic obstruction. Subsequent to PVP, the patient experienced recurrent macroscopic haematuria and pubic pain. Investigations confirmed the presence of a prostate-symphyseal fistula, a rare complication of PVP. We believe this to be the first reported case of successful treatment with robotic assisted radical prostatectomy.

## 1. Introduction

Photoselective vaporisation of the prostate (PVP) has become an established treatment for the lower urinary tract symptoms due to benign prostatic hyperplasia (BPH). A meta-analysis of randomised controlled trials has demonstrated equivalent functional outcomes to transurethral resection of the prostate but a complication profile in favour of PVP [1]. Prostatosymphyseal fistula formation is a rare complication of any form of transurethral prostate surgery for BPH and a literature search finds only eight reported such cases [2–5]. Whilst treatment has predominantly required surgery, we believe this to be the first reported case of treatment with robotic assisted radical prostatectomy.

## 2. Case Presentation

A 71-year-old man underwent PVP following a long history of lower urinary symptoms and failed drug treatment with tamsulosin. Preoperatively, the prostate had a volume of 44 cc as measured by transrectal ultrasound. Treatment was performed using the 180 W XPS GreenLight laser (American Medical Systems, Minnetonka, Minneapolis). The total operation time was 51 minutes and total lasing time was 25 minutes and 259 kJ of energy was expended. The operative procedure was uncomplicated and following surgery the duration of catheterisation and duration of hospital stay were 11 and 18 hours, respectively.

At one year following PVP, he presented with exercise related macroscopic haematuria. A urinary tract ultrasound and computerised tomography of the abdomen and pelvis failed to find an explanation for these symptoms. Rigid cystoscopic examination at that time appeared unremarkable. The patient also complained of perineal pain and bilateral groin pain on walking. A technetium 99 radionuclide bone scan revealed increased tracer uptake at the pubic symphysis with erosive changes likely related to symphysitis pubis and he was then referred for management by an orthopaedic surgeon.

Over the next 12 months, the patient continued to complain of urinary symptoms and haematuria. Over this time, he continued to experience bilateral groin pain on walking which was managed with analgesics. Empirical treatment with dutasteride failed to alleviate the urinary symptoms and a further rigid cystoscopic examination was performed. On this occasion, an actively bleeding ulcer was observed on the anterior aspect of the prostatic urethra and there appeared to be a calculus present within. Access and visibility were difficult due to the anterior location and the side firing Greenlight laser was therefore used to cauterise visible bleeding following extraction of the calculus.

**Figure 1:**
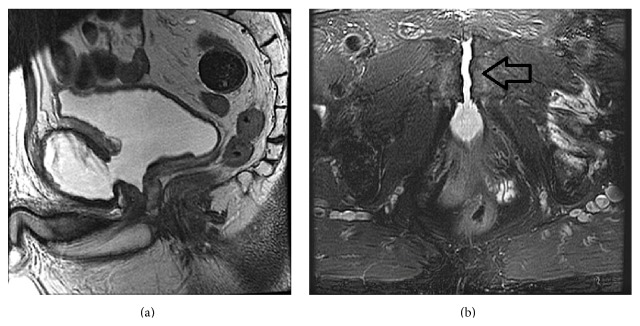
Images illustrate communication of anterior prostate and pubic symphysis. (a) is a midsagittal T2 weighted MRI without fat saturation. (b) is an axial T2 weighted MRI with fat saturation.

Magnetic resonance imaging of the prostate was then undertaken and this revealed the presence of a communication between the anterior part of the prostate and the pubic symphysis with urine filling the space of completely eroded symphysis pubis cartilage (Figure 1), confirming a diagnosis of a prostatosymphyseal fistula.

**Figure 2:**
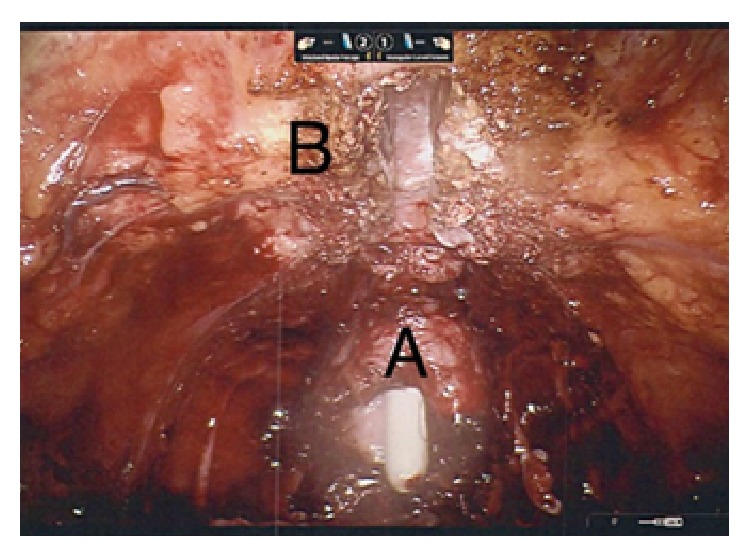
Operative view of prostatosymphyseal fistula. Following division of communication between the prostate and symphysis pubis, a large defect in the anterior prostate (A) is seen, exposing the urethral catheter. The symphysis pubis cartilage has been completely excavated (B).

Following a review of the literature, the decision was made to undertake treatment with robotic assisted radical prostatectomy. Using the Da Vinci Si robotic platform, surgery was performed using a six-port transperitoneal approach. There was surprisingly little difficulty in accessing the retropubic space. The endopelvic fascia was opened on each side and the prostate was found to be adherent to the symphysis pubis. Upon disconnecting the prostate flush from the pubic bone, a large defect in the anterior aspect of the prostate was uncovered and a clean defect was observed in the space where cartilage of the symphysis pubis had been completely eroded (Figure 2).

The patient made an uneventful recovery. Histopathology of the prostate specimen found incidental focal Gleason Score 3 + 3 = 6 adenocarcinoma.

At one month following procedure, the patient reported complete resolution of pain and had returned to playing golf. Urinary leakage was minor and necessitating a single pad per day.

## 3. Discussion

Prostatosymphyseal fistulas are rare, and as a result of symptoms being predominantly orthopaedic and infective, a delay in diagnosis is not uncommon. This patient concurrently had significant urinary symptoms, mainly manifested by recurrent episodes of macroscopic haematuria, and, in spite of initial full investigation, there was a failure to determine the diagnosis of prostate-symphyseal fistula. The symptoms and delay in diagnosis are not inconsistent with cases previously reported [2]. Of these patients, all eight presented with difficulty and pain when walking with vague groin and lower abdominal pain. Often patients are investigated with a CT scan which may show inflammatory changes to the pubic symphysis, osteitis pubis, which is then treated conservatively. Only when patients' orthopaedic and urological symptoms progress, is this investigated with further imaging and cystoscopy to reveal the fistula.

It is evident that initial cystoscopy had failed to diagnose the condition. When a 30-degree lens is used on a rigid cystoscope, visibility of anterior structures and especially in the anterior prostate can be impaired. Additional to this was a lack of index of suspicion that the orthopaedic and urinary symptoms were connected. In hindsight, a flexible cystoscope may better have clarified abnormalities in the anterior aspect of the prostate.

The pathogenesis of prostatosymphyseal fistulae may in some instances be unknown though there are hypotheses that it may be due to capsular weakening caused by prior radiotherapy and that the use of an indwelling catheter may lead to a bacterial infection with later spreads to the pubic symphysis [5]. In our case, we hypothesize that inadvertent capsular perforation at the time of initial PVP would likely have been associated with anterior urinary extravasation.

There is no standardised management of prostatosymphyseal fistulae. In a recently published review of prostate-symphyseal fistula, two were treated with radical prostatectomies and four were treated with open fistula repair using omental or peritoneal flaps whilst two were treated conservatively with catheterisation alone [2]. In two reports [2, 3], conservative management was the initial treatment approach but in all cases there was a failure of resolution and surgical repair then undertaken. The surgery described has required major open pelvic surgery but the availability of robotic surgery at our institution allowed for a minimally invasive approach. We opted for radical prostatectomy over reconstruction given that our familiarity with robotic prostatectomy as well as our concerns about adequate access to mobilisation of the omentum in the robotic environment was significantly greater.

## 4. Conclusion

Our case highlights the challenge of delayed diagnosis of prostatosymphyseal fistula and our first reported experience in its safe treatment with minimally invasive surgery in the form of robotic assisted radical prostatectomy.
